# Media analysis reveals the conservation risk of lost and active fishing gear in freshwater ecosystems of Hungary

**DOI:** 10.1038/s41598-026-43420-z

**Published:** 2026-05-15

**Authors:** Viktor Löki, Zsolt Neményi, Attila Hagyó, András Nagy, Zoltán Vitál, Tamás Malkócs, Balázs András Lukács, Attila Mozsár, Orsolya Vincze

**Affiliations:** 1 Wetland Ecology Research Group, HUN-REN Centre for Ecological Research, IAE, Bem tér 18/C, Debrecen, 4026 Hungary; 2 National Laboratory for Climate Change, HUN-REN Centre for Ecological Research, Karolina út 29, Budapest, 1113 Hungary; 3 Pál Juhász-Nagy Doctoral School of Biology and Environmental Sciences, University of Debrecen, Egyetem tér 1., Debrecen, 4032 Hungary; 4 Research Center for Fisheries and Aquaculture, Institute of Aquaculture and Environmental Safety, Hungarian University of Agriculture and Life Sciences, Anna-liget u 35, Szarvas, 5540 Hungary; 5 Littoral, Environnement Et Sociétés (LIENSs), UMR 7266, CNRS, La Rochelle Université, 2 rue Olympe de Gouges, La Rochelle, France; 6 HUN-REN Balaton Limnological Research Institute, Klebelsberg Kuno utca 3., Tihany, 8237 Hungary

**Keywords:** Anglers, Conservation, Fishing, Threatened species, Non-target species, Conservation biology, Freshwater ecology

## Abstract

Bycatch by fishing gear is a major cause of biodiversity loss. While bycatch is relatively well studied in marine ecosystems, there is limited information available on the frequency and the importance of bycatch in freshwater habitats, and the breadth of wildlife taxa affected. To explore this question, we conducted research based on online resources, including four social media platforms. We recorded 200 cases of fishing gear interactions with animals (excluding fish) between 1984 and 2024 in Hungary, along with information on the affected species and the location of the event. A total of 226 individuals of 64 animal species were affected by bycatch. Birds were the most frequently affected (60.6%), followed by reptiles (27.9%), while other taxa accounted for a total of 11.5% of records. The majority (72.6%) of the 226 bycaught individuals belonged to taxa protected by Hungarian law. The overwhelming majority of cases (n = 192, 96.0%) involved only two types of fishing gear: lines and/or fishing hook. Lost fishing gear was responsible for the majority of entanglements (n = 111, 55.5%), but actively used fishing gear was responsible for a comparable number of cases (n = 86, 43%). Our study highlights that bycatch from lost or active fishing gear poses a potential conservation concern requiring systematic investigation in freshwater ecosystems. Many of the affected taxa include species of conservation concern, underscoring the ecological significance of these interactions. In summary, our study highlights the conservation implication of bycatch in freshwater ecosystems, and calls for evaluating the true magnitude of its impact, especially in areas of high conservation importance.

**Supplementary Information:**

The online version contains supplementary material available at 10.1038/s41598-026-43420-z.

## Introduction

Bycatch—the capture of non-target organisms by unselective fisheries practices—is one of the most pressing nature conservation issues worldwide^1^. While the term bycatch is usually used in connection with fishing gear that is in active use, animals can be trapped, entangled in, or hooked by abandoned, lost or discarded fishing gear (hereafter ALDFG)^2^. In this study, we use the word bycatch to refer to both fishing gear in active use and ALDFG, and we specify the type when we only refer to one of them. The effect of bycatch on wildlife has been intensively studied in marine ecosystems, especially motivated by the loss of charismatic taxa, such as sea turtles and marine mammals^2^. Moreover, ALDFG is one of the major components of marine and seafloor litter^3^. The effect of bycatch on wildlife is, however, much less studied and understood in freshwater ecosystems^4^. Consequently, there is a significant lack of knowledge on the prevalence or the extent of conservation risk associated with bycatch in freshwater ecosystems globally^4,5^. Marine fisheries often span large areas of national or international waters while freshwater fisheries are of local scale thus the environmental impact of bycatch is difficult to compare between the two systems. Nevertheless, the knowledge gap may be represented by the disparity between the share of freshwater fisheries in global yields (11%) and the proportion of bycatch research focusing on freshwater fisheries (3%)^5^. Nonetheless, fishing gear is believed to represent a common type of litter in these ecosystems as well^6–9^.

Besides non-target fish species, turtles, mammals and birds can be affected by bycatch^2,5,10^. Sea-^11^ and freshwater turtles^12^ are among the most frequently recorded victims of bycatch, with numerous recorded suffocations, amputations, and other lethal lesions caused by fishing gear. Ingestion of fishing gear, especially plastic fragments mistaken for food, can cause intestinal blockages, leading to starvation or suffocation^13^. Besides turtles, mammals, especially marine mammals are often victims of bycatch: a recent review identifying forty different species of marine megafauna caught by ALDFG highlighted that marine mammals accounted for 70% of all bycatches around the globe^10^. Birds living in or near aquatic ecosystems are also at high risk of bycatch. Entanglement can impair their ability to fly, swim or search for food, while ingested hooks can cause internal injuries, infections and death^14^. Besides direct injury or immobilisation, lead (i.e. fishing sinkers) ingestion is a major issue in the case of birds^15,16^.

Citizen science initiatives engage volunteers in data collection and online resources to produce large data sets, which may complement traditional field-based research^17^. In addition to obtaining data through field monitoring programs, gathering information from online sources can be efficient, geographically widespread, and highly informative^18^. For example, analysing publicly available videos of recreational fishing may provide valuable ecological and social insights, support a better understanding of macroecological patterns^19^, aid the assessment and conservation of exploited species and contribute to the monitoring of recreational fisheries^19^. During the past few years, online resources, including social media platforms, have increasingly served as data sources used for hypothesis generation in research in many areas, and have proven especially informative in conservation sciences and in the monitoring of flagship species^20–22^. Social media has recently been highlighted as an informative data source also on the prevalence of metal-containing ALDFG in freshwaters, through the analysis of the catches of recreational magnet fishers^7^. A pioneering study analysing 33 Youtube videos identified the threat ghost fishing nets pose to freshwater ecosystems in Brazil, documenting the bycatch of 15 fish, four reptile and one bird species^36^. Another recent study on the impacts of ALDFG on Indian biodiversity also relied on on multiple social media platforms (Facebook, Twitter, YouTube and Google;^23^), documenting the bycatch of 35 species, including many listed as threatened by the IUCN. Yet, social media-based evaluations likely underestimate bycatch frequency, since charismatic animals are more likely to be reported. This work thus highlights the urgent need to evaluate the conservation risks posed by fishing gear in freshwater ecosystems.

Since the prevalence of bycatch and ALDFG, and their effects are highly understudied in freshwater ecosystems (see^4,5^), we conducted a social media analysis in Hungary (Central Europe) to (1) determine which non-target species (other than fish) are threatened by bycatch in freshwater ecosystems; (2) identify the types of fishing equipment responsible for the majority of events; (3) identify the types of water bodies that are most frequently exposed to bycatch.

## Materials and methods

### Social media analysis

To explore the frequency of bycatch in freshwater ecosystems, we performed systematic searches for online content indicating such events in Hungary. We explored a total of four key online platforms, namely Facebook (web 1): https://www.facebook.com/ [Accessed: 07/05/2024], YouTube (web 2): https://www.youtube.com/ [Accessed: 07/05/2024], Google (web 3): https://www.google.com/ [Accessed: 07/05/2024], and Arcanum (web 4): https://www.arcanum.com/ [Accessed: 07/05/2024], a Hungarian national database of digitized cultural and scientific content. The search engines of the above platforms were used to identify relevant content by entering the following keywords and their combinations in Hungarian: ‘beakadt’, ‘beleakadt’, ‘fennakadt’, ‘belegabalyodott’ (as different synonyms for entangled), ‘lenyelte (ingested)’ ‘horog’ (hook), ‘damil’ (line), ‘szerelék’ (tackle), ‘állat’ (animal), ‘madár’ (bird), ‘hüllő’ (reptile), ‘teknős’ (turtle), ‘sikló’ (colubrid snake), ‘béka’ (frog), ‘gőte’ (newt), ‘emlős’ (mammal). Our analysis did not include the anglers’ original target taxonomic group, fish. The search on each platform was repeated multiple times using various combinations (20+) of the above keywords, until no new records were detected. In total, more than 3000 posts were found with the combinations of these keywords that were later closely analysed for relevant content. Records were only retained when a direct contact between lost or active fishing gear and a live or dead animal was clearly visible on the content, and the interaction was active at the time of observation (~ 2700 posts excluded at this point). Duplicates (e.g., across platforms) were identified through visual similarity and overlapping information on date, location or surroundings and were excluded. Documented content where the implicated species could not be identified to at least genus level were excluded from the database. The final list of cases included 200 records: 132 were Facebook posts, 36 online newspaper articles, 13 blog entries, 6 YouTube videos, 10 digitized print media, and 3 printed and digitized grey scientific literature.

Documented content from social media platforms (Facebook and YouTube) was uploaded between 10 August 2011 and 20 March 2024, while Google and Arcanum content were dated between 21 February 1984 and 2 January 2024. The documented contents of interest (video, photo, and detailed descriptions) were carefully examined to determine (i) the type of bycatch: hooking (evident hook penetration in the mouth/beak) or entanglement (animals wrapped in fishing lines; in these cases no hook was visible in the footage, although their presence can not be ruled out entirely in most cases); (ii) the type of fishing equipment involved: active gear (bycatch observed during an active fishing event) or ALDFG (bycatch recorded independently of an active fishing event, regardless of when it occurred); and (iii) the identity of the affected species, classified to the lowest possible taxonomic level, ideally to species. All species were identified by VL, except for a few cases, which are listed in the Acknowledgments. Additionally, we geolocalized the recorded events based on the description or recorded the location provided by the content (n = 110), otherwise the location remained unspecified (n = 90). Finally, geolocalized water bodies found in the analysis were categorized according to habitat type into the following categories: ‘natural lake’, ‘oxbow lake’, ‘river’, ‘small watercourse’, ‘mine pit lake’, ‘fishing pond’, ‘canal’, or ‘not alongside water’. Of all categories, mine pit lakes, fishing ponds, and canals were considered as artificial, all others as natural freshwater habitats. Animals subjected to bycatch were categorised based on their protection status in Hungary as given in the “Register of Protected Natural Assets” managed by the Under Secretariat responsible for Nature Conservation of the Ministry of Agriculture of Hungary, available to search at: https://termeszetvedelem.hu/kereso/vedett-fajok/. The international protection status of species was accessed from the IUCN Red List^24^. The complete dataset analysed in this study is available in Supplementary Table S1.

### Statistical analysis

To explore the partitioning of individuals among habitat classes, types of fishing gear and protection statuses across taxonomic classes, we used Chi-square statistics. Post-hoc tests were conducted using the R package ‘rcompanion’^25^, using the pairwise Nominal Independence function with Benjamini–Hochberg method to control the false discovery rate due to multiple testing. All statistical tests were two-sided, and statistical significance was considered at a threshold of *p* < 0.05. While some of the variables might have interactive effects, here we only used single-predictor models due to the exploratory nature of this study, as well as due to the high collinearity among variables and the presence of missing data. For the graphical presentation of the taxonomic scale of animals affected by fishing gear, a phylogenetic tree was generated using the ‘rotl’ R package^26^, using the list of taxa as query. When an observation could not be identified to the species level, a species belonging to the same higher level taxon was used to build the phylogeny, and the branch was subsequently relabeled with the name of the higher level taxon.

## Results

### Bycatch across space, time and habitat types

**Fig. 1:**
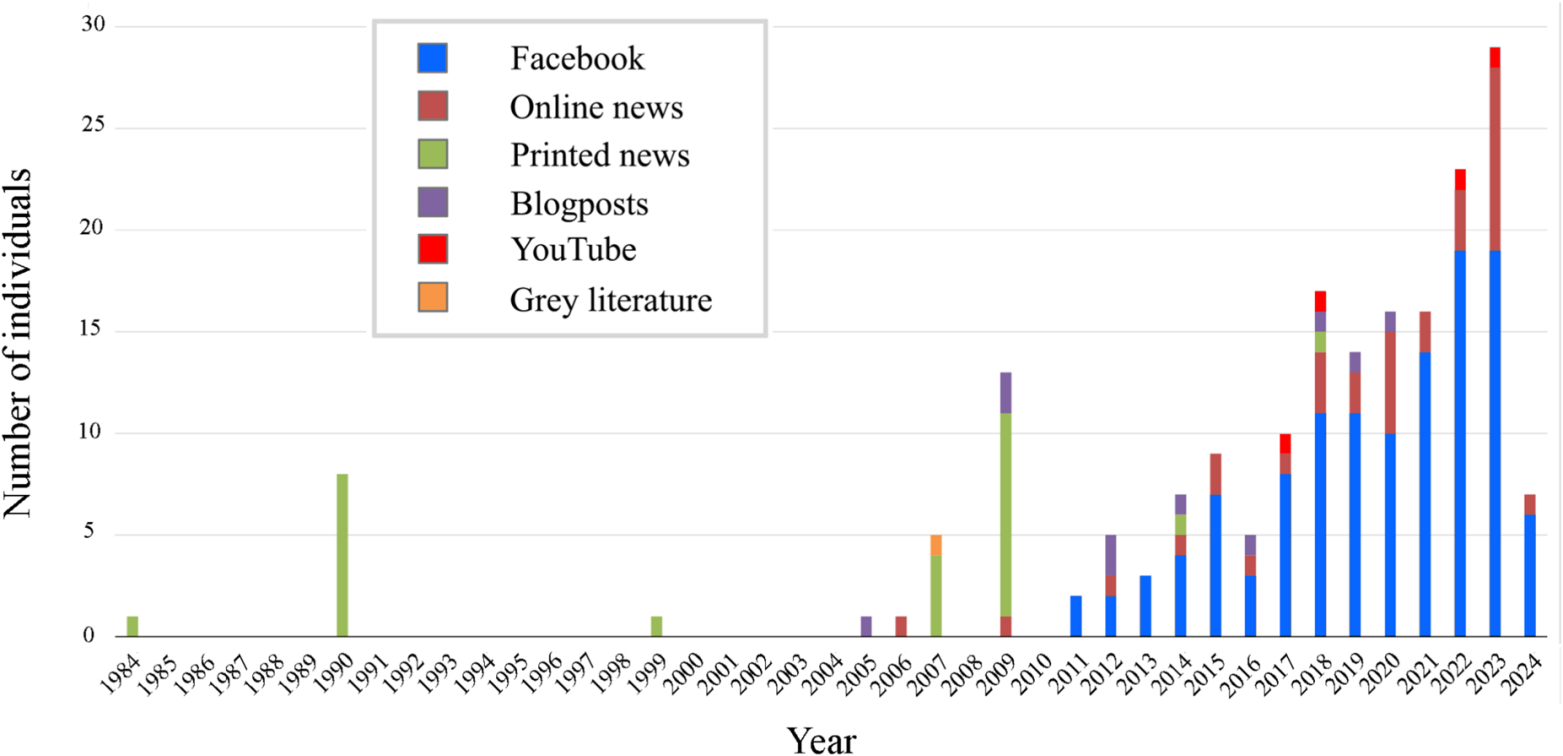
Number of entanglement cases in media sources between 1984 and 2024 in Hungary by year and media type, where the date could be determined from the post or article (n = 163). Note that 2024 represented an incomplete year during our media analyses.

**Fig. 2:**
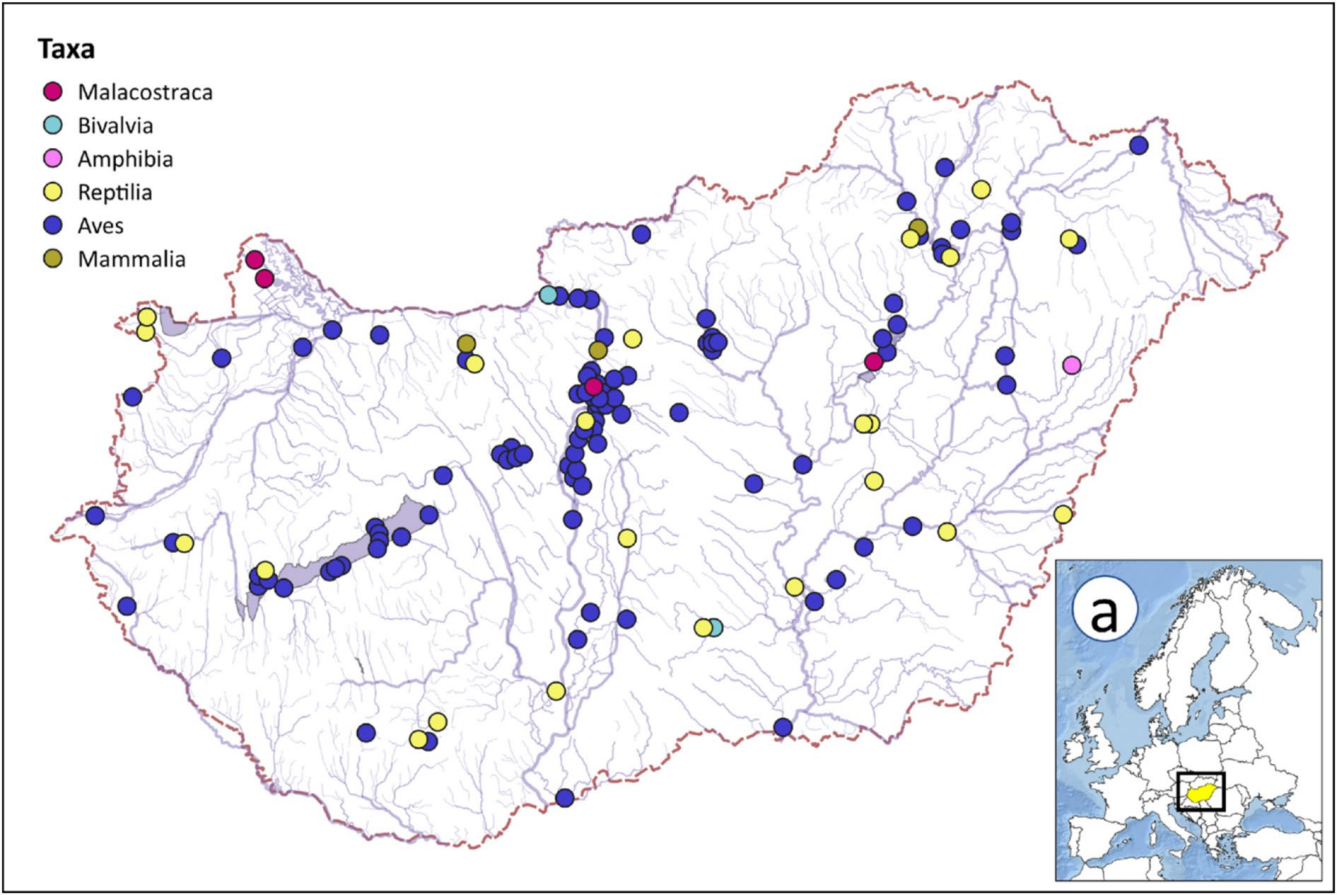
Spatial distribution of geolocalised entanglement cases recorded in Hungary (n = 110). The map was generated by Zoltán Vitál in QGIS software (v. 3.28, https://qgis.org/).

Our media analysis spanned forty years, documenting 200 cases of bycatch in Hungary between 1984 and 2024, some involving multiple individuals. Of these, 163 records were successfully dated based on information from the media sources (Fig. 1). Additionally, 110 of the 200 records were successfully georeferenced, also based on information provided by the media content (Fig. 2). Habitat classification could be completed both in case of the 110 georeferenced records, as well as for an additional 22 non-georeferenced records based on detailed evaluation of the media content. We expect no systematic spatial bias among the non-georeferenced cases, as their lack of location information is primarily due to missing or unclear reporting rather than the characteristics of the water bodies themselves. From 1984 to 2010, the number of recorded cases was generally low, with only sporadic incidents reported, except for a few peaks in reported cases in 1990 (8 cases), 2007 (5 cases) and 2009 (13 cases). From 2011 onward, in parallel with the rise of social media use, there’s a noticeable increase in the frequency of reported cases, although with fluctuations. Starting in 2015, there is a marked and consistent upward trend in the number of records, peaking in 2023 with 29 cases.

There were 132 cases where the habitat type could be identified. More bycatch records came from natural (n = 78, 59.1%) than from artificial (n = 43, 32.6%) aquatic habitats. Of the categorised habitat types, bycaught animals were most commonly found alongside rivers (n = 36, 27.3%), followed by natural lakes (n = 27, 20.5%), fishing ponds (n = 15, 11.4%), artificial lakes (n = 12, 9.1%), and mine pit lakes (n = 11, 8.3%). Less affected habitat types were oxbow lakes (n = 8, 6.1%), small watercourses (n = 7, 5.0%) and canals (n = 5, 3.78%). A total of 11 bycatches (8.3%) came from non-aquatic habitats.

### Taxonomic groups, survival rate and conservation status

**Fig. 3:**
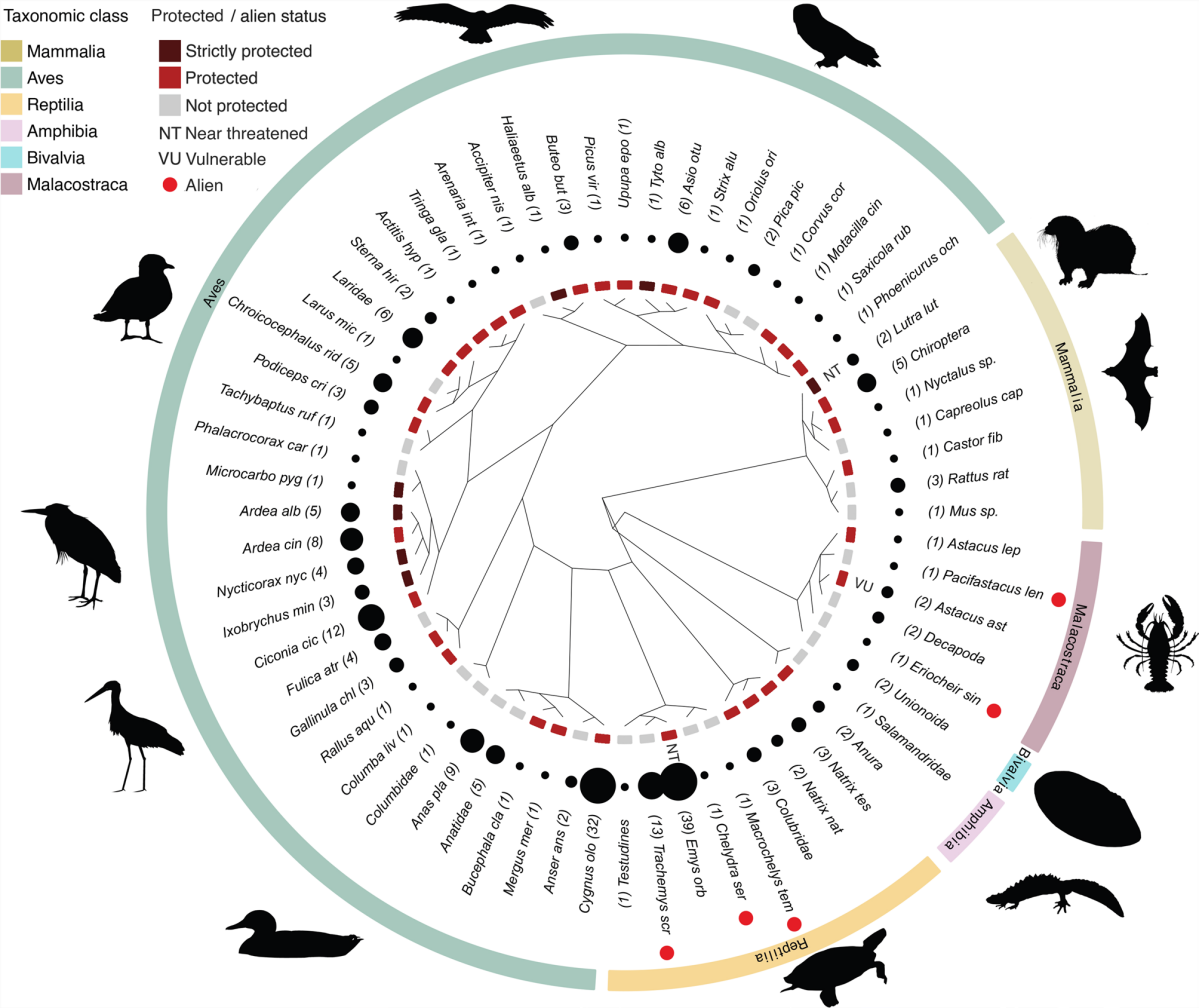
Bycaught animal groups in Hungary (including species, number of individuals and conservation status). The size of the black circles corresponds to the number of affected individuals. IUCN categories are indicated with their two-letter abbreviation, except for Least Concern, which is not indicated.

**Fig. 4:**
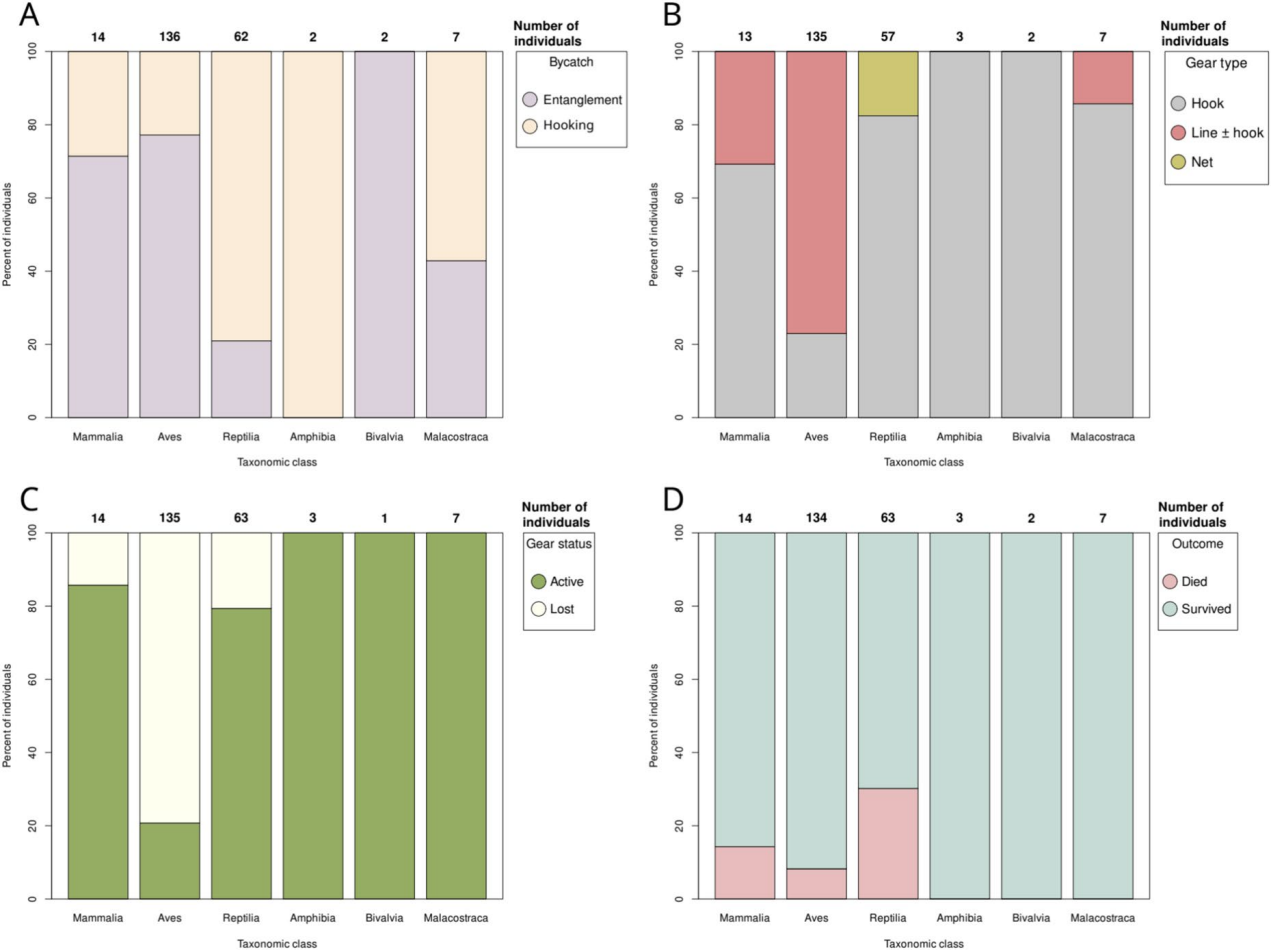
Percentages and affected individuals of entanglement (**A**) and gear type (**B**), gear status (**C**) and outcome of the entanglement of bycatch (**D**). Sample sizes vary among the subfigures depending on the number of individuals where the entanglement, gear type or status or the outcome could be extracted from the media records.

**Fig. 5:**
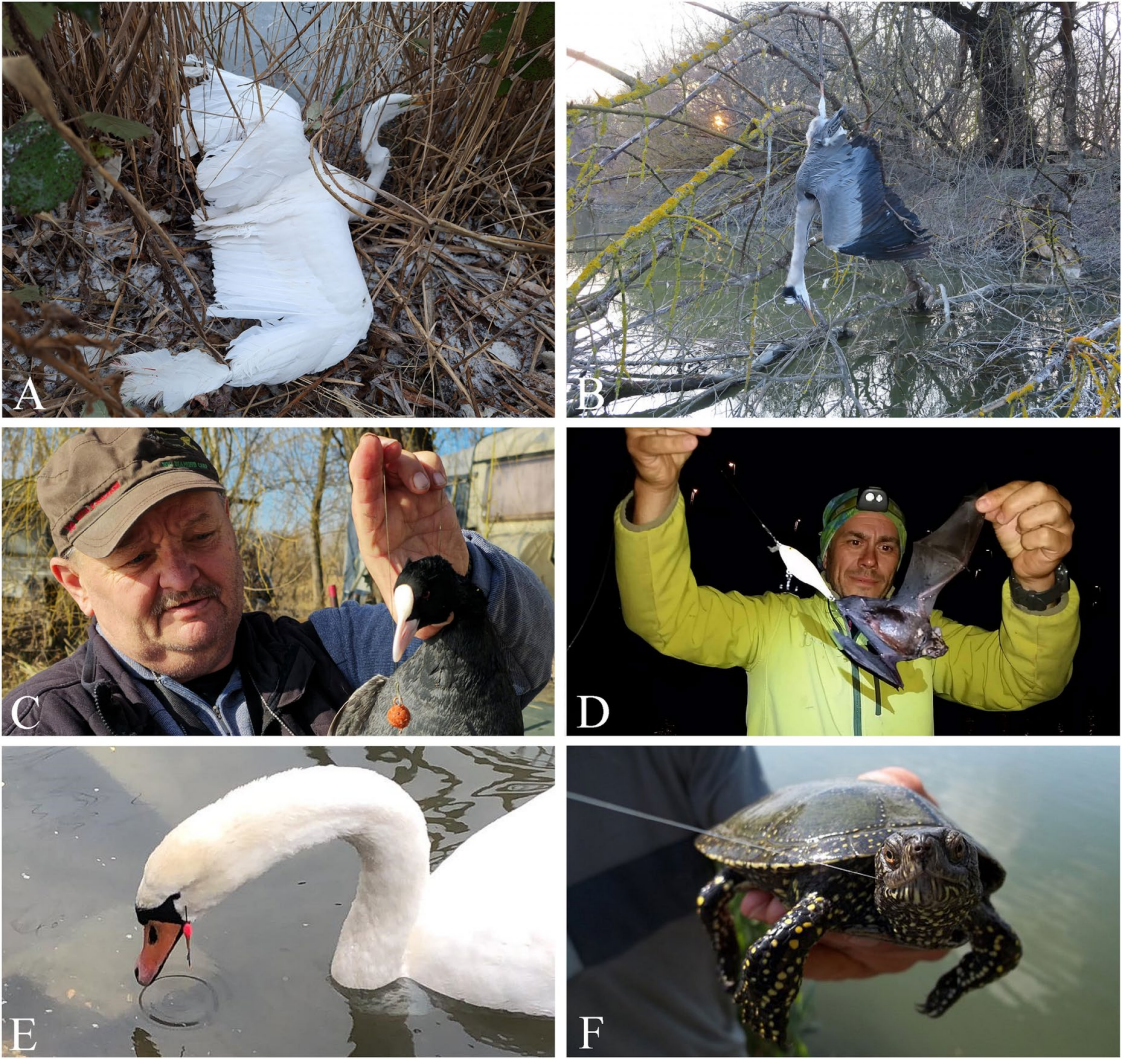
Some typical examples of animal entanglement with ALDFG or bycatch in Hungary. (**A**) an entangled alive individual of *Ardea alba*; (**B**) a deceased individual of an entangled *Ardea cinerea* (**C**) a bycaught individual of *Fulica atra* hooked by bait during fishing; (**D**) a rare bycatch, an individual of *Nyctalus sp.*; (**E**) the most common entangled bird species and species overall was *Cygnus olor* with a total of 32 records; (**F**) the most common reptile and the second most common bycaught animal was *Emys orbicularis* with 18 records. Image credits: (**A**) Belső tó – Hatvan, Hungary (by dr. Bálint Bártfai); (**B**) Dávid Balázs; (**C**) György Juhos; (**D**) László Hidvégi; (**E**) Tollas Barát Madármentés; (**F**) Ottó Boltizár.

A total of 226 reported individuals were affected by bycatch, representing a minimum of 64 taxa, of which 54 were identifiable at the species level with 199 individuals (Fig. 3). The overwhelming majority of the bycaught individuals were birds (n = 137, 60.6%). The second most affected taxonomic group was reptiles with 63 individuals (27.9%). Mammals, decapods, amphibians, and molluscs were less often reported to be affected by bycatch, with 14, 7, 3 and 2 individuals, respectively. Of all records, 191 (85.7%) individuals were reported to have survived the bycatch event and only 32 (14.3%) individuals deceased. The fate of an additional seven individuals could not be determined (Fig. 4). Some typical examples of bycatches are shown in Fig. 5.

Of the 226 bycaught animals, 164 (72.6%) are currently protected at the species level by Hungarian law, including some strictly protected taxa, such as 15 individuals of six bird species (*Ardea alba, Haliaeetus albicilla, Ixobrychus minutus, Microcarbo pygmeus, Nycticorax nycticorax, Tyto alba*) and two mammalian individuals (*Lutra lutra*). Within our database, 43 bycaught individuals are classified as near threatened or vulnerable by the IUCN Red List: *Emys orbicularis* (39 individuals), and *Lutra lutra* (2 individuals) are listed as near threatened, while *Astacus astacus* (2 individuals) is listed as vulnerable. 131 individuals are currently categorized as Least Concern by the IUCN. Alien species are represented by 17 bycaught individuals of six taxa (four turtle species and two decapods).

### Types of bycatch

The type of gear status (active vs. ALDFG) could be identified in 197 cases involving 223 animals. More than half (54.7%, 122) of the bycaught individuals within our database were impacted by ALDFG rather than actively used fishing equipment, but major differences were detected across taxonomic classes (χ^2^ = 84.88, df = 5, *p* value < 0.0001). For instance, all amphibians, bivalves and crustaceans and the majority of mammals and reptiles (85.7% and 79.4%, respectively) were caught by actively used fishing gear, while over 79.3% of birds were entangled in and/or hooked by ALDFG (Fig. 4).

The type of gear was identified in 193 cases involving 217 individuals. The overwhelming majority of bycaught animals encountered two gear elements, namely hooks (45.2%) and lines with or without hooks (50.2%, see also Fig. 4). Only 4.6% of the individuals recorded here were involved in interactions with fishing nets (Fig. 4). Post-hoc tests revealed significant pairwise differences only in the case of birds, which had a higher probability of being bycaught by ALDFG than mammals (*p* < 0.0001), reptiles (*p* < 0.0001), amphibians (*p* = 0.0137) or crustaceans (*p* < 0.0001).

The type of bycatch (i.e. entanglement vs. hooking) could be identified in 197 cases involving 223 individuals. The overwhelming majority of bycaught animals (59.6%) were entangled, rather than hooked, but major differences were detected in this regard across taxonomic classes (χ^2^ = 61.89, df = 5, *p* value < 0.0001; Fig. 4). For instance, the majority of mammals and birds, as well as all bivalves were entangled in lines with or without hooks, while the majority of reptiles, crustaceans, as well as all amphibians were hooked. Animals from certain taxonomic classes were more likely to experience specific types of interactions with fishing gear. Mammals were more frequently entangled than reptiles (*p* = 0.0015). Birds were more prone to entanglement than reptiles (*p* < 0.0001) and amphibians (*p* = 0.0416).

Regarding the fate of the entangled animals, only 14.3% (32 individuals out of 223 where the outcome could be established before release) deceased as a consequence of the entanglement. The proportion of animals that died in each taxonomic class differed (χ^2^ = 18.93, df = 5, *p* value = 0.0020). More specifically, death was only recorded in mammals, birds and reptiles, with the highest death rate in the case of reptiles (30.2%), followed by mammals (14.3%) and birds (8.2%). No death was recorded in amphibians, bivalves or crustaceans, but the number of individuals with identified outcomes was also very limited in these taxonomic classes. The difference in the proportion of deaths was only statistically significant between birds and reptiles (*p* = 0.0008, Fig. 4).

## Discussion

Our online media survey, presenting data from the last four decades, points out the threats to freshwater biodiversity posed by bycatch by both active and lost fishing equipment. Altogether, 64 animal taxa, including birds, mammals, reptiles, amphibians and macroinvertebrates, were affected by bycatch in our survey. We documented a high ratio of protected taxa among the affected animals, which underlines the potenital need for conservation measures in freshwater ecosystems to reduce the adverse effects of bycatch on biodiversity, similarly to marine ecosystems^23^. Importantly, our findings highlight the urgent need for systematic, field-based surveys to accurately assess the true extent of fishing gears’ impacts on freshwater wildlife. Especially, since online-media based analyses are likely to underestimate the frequency and severity of bycatch events. We recommend the adoption on preemtive measures, especially in areas with high angling pressure and/or high conservation importance. These could include (i) regular removal of ALDFG and active monitoring to prevent accumulation of fishing gears that pose risks to birds, mammals, and fish. (ii) Highly recommended use of wildlife-safe fishing gear (e.g., biodegradable hooks, sinking lines to reduce bird strikes) to reduce injury and mortality of non-target species. (iii) Bycatch mitigation programs, including angler training on safe release methods and the use of gear modifications proven to decrease incidental capture of protected species.

In marine habitats, animals are most frequently entangled and trapped by fishing nets and ropes^23^. In contrast, based on our study, fishing lines with or without hooks were responsible for most bycatch events in the freshwater ecosystems of Hungary. This difference is explained by the dissimilarities in fishing practices between marine and inland fisheries and the peculiarities of Hungarian fishing regulations. The former serves commercial aims and requires the use of large nets. Contrary, freshwater fisheries in Hungary are dominated by angling, which uses hooks, lines, and rods for capturing, primarily for recreational purposes. Due to the regulations of the fisheries in the study area (i.e., commercial fishing and the use of nets is prohibited in Hungary since 2016), our study may underestimate the role of abandoned nets and ropes in bycatches in freshwater ecosystems outside Hungary (c.f.^4^). Our findings, however, clearly show that animals can be entangled by abandoned gear and tackle derived from recreational angling.

The reliable assessment of both the frequency of bycatch events and vulnerability of a given taxon is affected by the size and behaviour of the entangled animals and survivorship bias. Small taxa were underrepresented in our dataset of bycaught animals, while, for instance, the mute swan (*Cygnus olor*) was one of the most frequently bycaught bird species. It remains to be determined whether small animals are less prone to be affected by bycatch or they are less likely to be detected (e.g. shorter survival following bycatch, lower visibility, etc.) or reported^27^. Moreover, large body size, bright coloration and the use of anthropogenic habitats by bycaught animals might play a role in increasing detectability, while certain factors, such as charisma, might increase the likelihood of reporting the event^28^. Moreover, we suspect that differences of activity levels among species may affect how they behave in the event of bycatch, causing bias in the probability of being observed or recoded. For instance, nocturnal or cryptic mammals might be more likely to hide following bycatch, significantly decreasing detection probability. Furthermore, a moving entangled animal is generally easier to observe than a deceased, hidden, non-moving individual. For instance, the large size of mute swans allows them to move and survive long after entanglement, which could contribute to the high number of bycatch cases in this taxon.

In our study, fewer than 10% of bycaught individuals were recorded as deceased. While this may seem low, the figure likely underrepresents the true mortality rate, as observers often released entangled animals, and there was no post-release monitoring to assess long-term outcomes. Survival rates for undetected bycatches—particularly in remote or less monitored areas—are likely much lower, with delayed mortality or sublethal effects such as injury, impaired mobility, or increased predation risk remaining unaccounted for.

It is important to distinguish reported mortality from actual biological impact. Our dataset captures only visible, immediately documentable outcomes at the time of observation while overlooking broader ecological consequences. Thus, the seemingly low mortality rate should be interpreted with caution, as the true conservation impact of freshwater bycatch likely extends beyond immediate deaths, and includes indirect effects on individual fitness and long-term population viability.

Additionally, our research may be affected by data limitations and reporting biases. While social media offers a novel source of information on wildlife-fishing gear interactions, visibility bias may favour larger, more charismatic, or easily photographed species, underrepresenting smaller or less conspicuous taxa. In particular, the apparent dominance of birds, reptiles, and protected species in our dataset is likely influenced by reporting bias. These groups are often larger, more visible, and perceived as more charismatic or socially valued, increasing the likelihood that entanglement or hooking events involving them are photographed and shared online. In contrast, less conspicuous or charismatic taxa, such as amphibians, small mammals, or non-protected bird species, are probably underrepresented despite potentially experiencing similar interactions with fishing gear. This bias may therefore overestimate the relative importance of certain taxa while underrepresenting the broader taxonomic scope of the problem. Recognising this limitation is essential for interpreting our results and underlines the need for complementary, systematic field studies that can better capture these overlooked interactions. Variation in species detectability across taxa and environments, along with human tendencies to report more dramatic or emotionally charged events, may further skew the dataset.

Our study highlighted that animals entangled in fishing gear can be found deceased far from aquatic habitats, e.g., owls, white storks, and other birds (11 affected individuals, 5.5% of all records, of which nine birds). The most frequently affected species was the white stork (*Ciconia ciconia*, four times), which we hypothesize to have accidentally carried lost fishing gear into its nesting material. During other records (seven cases), mostly animals in private gardens were recorded in such events. This highlights that at least some animals might move away from water bodies, making a targeted survey on the effect of fishing gear difficult. Moreover, these observations suggest that displaced fishing gear might potentially affect species not associated with aquatic habitats.

Moreover, our dataset might also be biased spatially and especially temporally. The temporal and spatial distribution of the recorded bycatch events is likely influenced by uneven reporting effort and the rise of digital platforms, particularly social media, since around 2011. Although our dataset spans from 1984 to 2024, the frequency of documented cases has increased sharply in the past decade, coinciding with greater internet access, smartphone use, and the proliferation of social media worldwide, including Hungary. This likely reflects improved reporting capacity rather than a major increase in bycatch incidences. Although we did not stratify our survey seasonally, it is important to note that reporting rates are most likely higher during summer months, when fishing effort is the highest. Spatially, records are biased toward more populated or recreational areas, where both fishing activity and human presence are higher, and therefore reporting is more likely. As such, our findings should be interpreted as a conservative estimate of the true extent of bycatch events, influenced by the accessibility and visibility of both the events and the observers.

In the marine environment, practical solutions like physical marking—using tags and buoys—can reduce the chance of losing nets and traps or retrieving lost ones^29,30^. In freshwater ecosystems, however, similar markings are not feasible if bycatches are caused by hooks and fishing lines. Recognising the threats of bycatches, some inventions and products are already on the market, such as: more degradable (made from carbon steel instead of stainless steel), barbless, and specially shaped (e.g. circle) hooks, and devices for closed storage of waste line. Although most of these products are available in Hungary, only barbless or micro-barbed hooks are used frequently, especially for catch-and-release fishing. Although all of these are aimed to prevent and mitigate the adverse effects of bycatch and can be applied also in freshwater ecosystems, dissemination campaigns are necessary to promote their use. Accordingly, education and dissemination is critical. The problem of bycatch in marine ecosystems is widely educated and the majority of anglers are concerned about marine litter, they recognize their responsibility and are willing to contribute to waste avoidance and mitigation^31^. Similarly, raising awareness among anglers and recreational fishers on the risk posed by lost, discarded and abandoned fishing gear can help to reduce the amount of fishing gear litter in freshwater ecosystems. However, a Central European case study concluded that Instagram users who have personal knowledge about nature protection, do not want social media to educate them about nature-protection-related topics and do not consider Instagram a good source for such information^32^. Consequently, dissemination using the offline space might prove more beneficial, and environmental education campaigns aiming to sensitizing anglers to the issue of bycatch should focus on information boards, school-based activities and/other written material (e.g. dedicated newspapers, angling clubs, protected area visitor centers).

Our results confirmed that online media, especially social media, is an emerging tool for both research and data collection. The analysis of posts on Facebook^33,34^ or data mining of videos published on YouTube^19,35,36^ or the combination of these and other types of social and traditional media^23^ are good examples of how digital media offers a great potential for science to explore some questions. Data collection in the form of field studies and surveys is rather time-consuming and resource-demanding, however, by using the correct keywords on social media, we can build a local or even global dataset relatively quickly.

While social media-based data collection is a valuable tool for exploratory and pioneer studies, it carries inherent limitations, including spatial, temporal, and taxonomic biases in reporting. Therefore, we need to highlight that while data derived from social media analysis is indeed useful for generating hypotheses, it is not suitable to test them due to the limitations mentioned above. While this dataset provides useful insights into patterns of angling activity, its resolution and coverage are not sufficient to reliably identify priority areas for targeted conservation measures, but it can be useful to select areas for increased surveillance. Likewise, the current data do not allow robust estimation of bycatch rates per fisher or per unit waterbody area, or the population-level impact of bycatch, although such analyses could be pursued with more comprehensive monitoring. Strengthening data collection in future studies would enable more precise spatial targeting of interventions aimed at reducing bycatch and mitigating the impacts of lost or discarded fishing gear. To address these shortcomings, future research should aim to complement social media data with systematic field monitoring and citizen science initiatives that follow standardized reporting protocols. These protocols could involve standardized forms, possibly paired with QR code submissions at fishing sites, or in collaboration with fishing associations. Also, providing guidelines for recording evidence, such as proposing a simple gear-type taxonomy, guidelines for photography would aid the formalization of these datasets. Another major step in the formalization of citizen-science based datasets is the recommendation of minimal metadata fields, such as date, location coordinates, gear status, and outcome. Citizen science initiatives could play a key role in improving data quality by engaging recreational fishers in systematic reporting of catches, bycatch, and lost gear. Such participatory monitoring could not only expand spatial and temporal coverage of observations but could also foster a sense of stewardship, increasing awareness of the ecological impacts of angling practices. Involving fishers directly in data collection and interpretation may also enhance compliance with conservation efforts and encourage adoption of more sustainable fishing practices. Additionally, studies assessing the long-term outcomes for entangled individuals are crucial for understanding real impacts. Expanding the geographic scope to other freshwater systems and integrating fisheries data—such as fishing effort, gear types, and catch locations—could further support the development of targeted and effective conservation measures by helping to identify high-risk areas and periods for bycatch events.

## Supplementary Information

Supplementary Information.

## Data Availability

The datasets used and/or analysed during the current study available in Supplementary Table S1.
